# Enhanced Temporal but Not Attentional Processing in Expert Tennis Players

**DOI:** 10.1371/journal.pone.0002380

**Published:** 2008-06-11

**Authors:** Leila S. Overney, Olaf Blanke, Michael H. Herzog

**Affiliations:** 1 Laboratory of Cognitive Neuroscience, Brain Mind Institute, Ecole Polytechnique Fédérale de Lausanne (EPFL), Lausanne, Switzerland; 2 Laboratory of Psychophysics, Brain Mind Institute, Ecole Polytechnique Fédérale de Lausanne (EPFL), Lausanne, Switzerland; Istituto di Neurofisiologia, Italy

## Abstract

In tennis, as in many disciplines of sport, fine spatio-temporal resolution is required to reach optimal performance. While many studies on tennis have focused on anticipatory skills or decision making, fewer have investigated the underlying visual perception abilities. In this study, we used a battery of seven visual tests that allowed us to assess which kind of visual information processing is performed better by tennis players than other athletes (triathletes) and non-athletes. We found that certain time-related skills, such as speed discrimination, are superior in tennis players compared to non-athletes and triathletes. Such tasks might be used to improve tennis performance in the future.

## Introduction

In many sports, such as in tennis, excellent visual skills are necessary. From the 1950s on, a popular standpoint, advocated by optometrists, was that successful athletes are endowed with superior visual systems [1]–[3]. Optometrists assumed that any improvements in vision achieved through training would transfer automatically to improved sports performance. However, it was shown that specifically optometric tests such as visual acuity do not improve with training in general [4]. In sports research, Abernethy and Wood [5] showed that visual acuity and stereopsis in a pre-post training paradigm did not lead to improvements in optometric vision beyond those resulting from familiarity.

In contrast to the optometric view, more recent studies carried out in field hockey [6], snooker [7], and soccer [1], [8] suggest that “perceptual and cognitive factors” change with training [9]. These changes seem to occur on a long term basis. Since decades of training are generally necessary to become an expert in a particular sport, the performance of expert athletes is usually compared to that of novices who have rather short term training. Studies comparing experts' to novices' performance have investigated skills such as anticipation of opponents' intention based on partial information or advance cues [10], [11], visual search strategies [1], [12], [13] or recognition and recall of typical patterns of play from memory [6], [14], [15]. These studies consistently showed that experts performed better than novices.

Most of these studies used stimuli related to the domain of expertise [6], [16]–[23]. For example, tennis players' visual anticipation skills were tested with videotapes of expert tennis players performing either serves or ground strokes [24]. Expert players were found to be more accurate and faster in anticipating ball direction.

Only few studies related to perceptual or cognitive factors have used stimuli unrelated to the sport in question. Buckles, Yund and Efron [25], for instance, used a target detection task involving a group of patterns (such as vertical and horizontal stripes) and found an advantage for tennis players compared to non-players. Other studies used fixation and saccadic tasks and showed an advantage of elite shooters compared to non-shooters [26], [27].

In our view, the involved mechanisms in all these studies were described as perceptual as well as cognitive without making an actual distinction between them. We feel that it is quite important to dissociate perceptual from cognitive mechanisms. According to us, perceptual mechanisms are tested with low-level visual tasks such as vernier acuity or coherent motion detection using random dot kinematograms, which are higher-level mechanisms when compared with optometric functions [2], [3], [5] but lower-level mechanisms when compared with cognitive tasks which involve mechanisms such as recognition or anticipation [6], [14], [15].

In the present work, we used the same approach as that of perceptual or cognitive studies. We assumed that certain perceptual skills improve in tennis players during their long-term training (usually for decades) whereas others do not. Our goal was to identify basic visual *perceptual* skills that change versus those which do not change. For this purpose, we used a *systematic* study involving basic visual tasks that are beyond the simple visual acuity tasks (optometry) but not as specific as the usual sport-specific tasks (using domain-specific information). More specifically, the question addressed here was whether playing tennis only changes specific aspects of visual perception related to tennis (as shown by previous research mentioned above), such as tennis ball speed estimation, or whether more fundamental skills, such as coherent motion detection, are also improved. Again, by “fundamental” or “basic” visual skills we do not mean “optometry”. Rather, the tasks we were interested in were basic visual perceptual tasks that are known to improve with training (as revealed in other contexts) but which are not directly related to a particular sport.

We chose to study tennis players because tennis is one of the most popular sports [28] and, therefore, athletes were easily accessible. Most studies usually compare elite versus novice players of a same sport. Here, we compared the performance of skilled tennis players to athletes performing another sport (triathletes) and non-athletes (as estimated by a questionnaire). This was done in order to make sure that any potential differences could be linked to tennis (or at least racket sports) *per se* and not just any sport or a better physical shape. A battery of seven tests was developed, each of them being related to a particular aspect of visual perception. For this purpose, we selected several perceptual tasks in order to investigate whether there are fundamental perceptual skills for which tennis players perform better than controls.

Since tennis players need to react in a fast moving environment, we selected a coherent motion task [29] as well as a speed discrimination task [30]. A backward masking task was used to test performance under strong time constraints [31]. Since tennis players may need to quickly detect the tennis ball, two object detection tasks were used: one related to tennis (ball detection task; [17]) and one unrelated to tennis (pattern detection task; [25]). Further, the attentional blink paradigm requires processing a stimulus embedded in a stream of complex stimuli [32], which might reflect the complex environment of a tennis game from which players need to pick up the relevant information. Finally, the flash-lag effect [33] was used because it requires motion extrapolation which can be related to anticipation of the ball trajectory.

## Methods

### Subjects

Eighteen experienced tennis players, 18 experienced triathletes and 19 non-athletes participated in the study. Tennis players and triathletes were national competitors. Triathletes were chosen as a control group because they share a similar level of fitness but do not need fine spatio-temporal resolution as tennis players. The non-athletes were people who had never played any sport regularly (i.e., only occasionally would they participate in a sport game with friends) and had never tried playing tennis. All subjects were males, aged between 21 and 38 (mean = 28.5±6.2 years) and had normal or corrected-to-normal vision. Visual acuity was tested by means of the Freiburg visual acuity test [34]. Only participants who reached a value of 1.0 (corresponding to 20/20) for at least one eye, took part in the experiments. All observers gave their written informed consent to participate in the study and were paid for their participation. The experiment was approved by the local ethical committee (Lausanne University).

### Stimuli and procedure

Before starting the visual tests, a questionnaire was given to all participants, which established their amount of training, their level of fitness, and their ranking (for tennis players). This was mainly done to ensure homogeneity in each group of subjects.

The battery included seven low level visual discrimination tasks involving different visual aspects such as motion, attention, and temporal processing. Each of the tests will be described separately. All tasks and stimuli were presented on the same CRT (Cathode-Ray Tube) monitor (ViewSonic G90f+, screen size adjusted to approx. 34×26 cm, spatiotemporal resolution as mentioned below). All subjects were administered the seven tasks in a different order. They sat at a distance of 1 meter from the screen. Before each test, the task was explained to them and 10 practice trials were presented. Observers held two push buttons, one in each hand. These buttons were counterbalanced between subjects. Subjects could take breaks in between each test if they wished. The total duration of the tests battery lasted between 2 and 2.5 hours.

As a matter of consistency across all tasks, whenever data-points fell outside the mean±2SD range, these outliers (data-points) were excluded from the analysis.

#### Coherent motion

**Figure 1:**
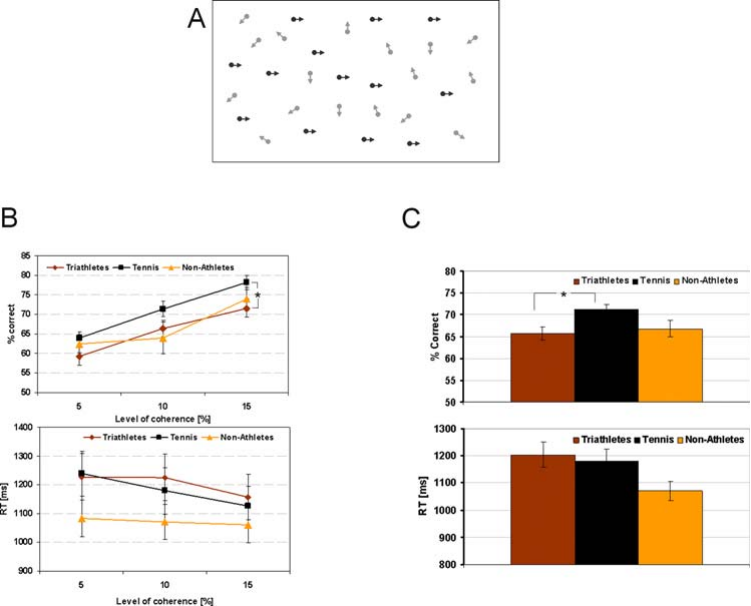
Coherent motion. A: Stimuli of the coherent motion task: In the example shown, the fraction of dots moving coherently to the right are shown in dark for clarity purposes. In the experiment, all dots had the same luminance. B: Results of the coherent motion task: Accuracy is shown in the top panel and reaction times are shown in the bottom panel as a function of coherence level. Error bars show standard errors. C: Results of the coherent motion task with all coherence levels collapsed: Accuracy is shown in the top panel and reaction times are shown in the bottom panel for each group of subjects. Error bars show standard errors.

*Stimuli*: Random dot kinematograms (RDKs) were displayed on the ViewSonic G90f+ with 1024×768 pixels, 100 Hz. RDKs comprised a rectangular patch containing 300 randomly arranged white dots (100 cd/m^2^) on a black background. Dots were positioned with 1/10 sub-pixel precision while applying antialiasing techniques, and positions were updated at frame rate. The dimensions of the patch of dots were 12×8°. Dot size was 1.8′ and dot speed was 5°/sec. Each dot had a limited lifetime of 100ms after which it disappeared and reappeared at a random location within the stimulus patch. A fraction of the dots were moved coherently, i.e., in one direction, either to the left or to the right, in the field of randomly moving dots (Figure 1A). There were 3 levels of coherence: 5%, 10% and 15%.

*Procedure*: There were a total of 120 trials, i.e., 40 trials for each level of coherence. Stimulus duration was 200 ms. The direction of motion and the level of coherence were presented randomly. Subjects had to determine the direction of coherent motion, either to the left or to the right.

*Statistical methods description:* Accuracy (percent correct values) and reaction times (RTs) were measured. Accuracy and RTs performances of the three groups of subjects were compared by means of a one-way analysis of variance (ANOVA) for each level of coherence (5%, 10%, and 15%).

#### Speed discrimination

*Stimuli*: Stimuli were displayed on the ViewSonic G90f+ with 1280×1024 pixels, 75 Hz. Random dot kinematograms (RDK) were used to simulate radial (contracting and expanding) and rotational optic flow. Each RDK contained a uniform distribution of 190 white motion dots displayed on a low luminance grey background (5.2 cd/m^2^). Dots were positioned with 1/10 sub-pixel precision while applying antialiasing techniques, and positions were updated at frame rate. All dots were presented in an annular field of 24° diameter (no dots presented in the central 4°). Each dot moved through a radial speed gradient, the mean speed (21.3°/sec) of which could be varied between tests in a direction consistent with the type of complex motion (expansion, contraction or rotation). The motion of the dots in the RDK was determined as though the trajectory of each dot had been first calculated in the continuous-time domain. The position of each dot in each frame of the RDK was then obtained by sampling from the continuous time trajectory at discrete time intervals corresponding to the refresh time of the screen. This was necessary because, in order to maintain a constant global velocity field in an optic flow stimulus, the motion of each local feature in the stimulus must accelerate. For example, in a pattern expanding at a constant velocity, each feature accelerates centrifugally such that its speed is proportional to its distance from the focus of expansion. We calculated dot positions by sampling from continuous-time trajectories in which the dots were accelerating at all times. In this way, we were able to match the speeds of different types of complex motion stimuli precisely ([30] for more details). The dots' diameter was 10′ and their lifetime was 156 ms to reduce motion discrimination based on the trajectories of single dots.

**Figure 2:**
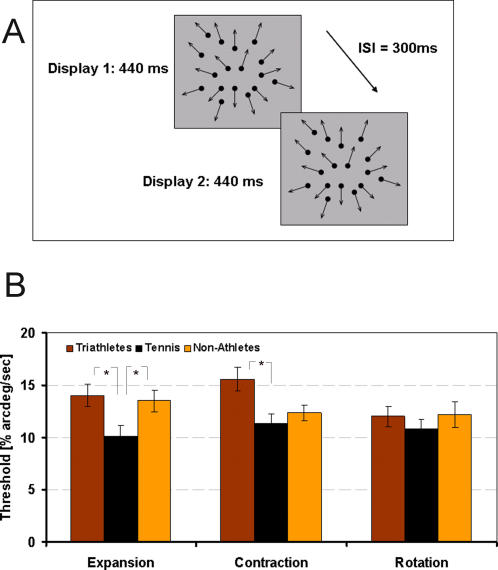
Speed discrimination. A: Stimuli of the speed discrimination task: In the example shown, the dots are in expansion for both stimuli which are presented for 440±40 ms with a 300 ms inter-stimulus interval. Subjects had to determine which of Display 1 or 2 was going faster. B: Results of the speed discrimination task: The mean threshold is shown for each optic flow type and for each group of subjects. Error bars show standard errors.

*Procedure*: Subjects performed a two-interval forced-choice (2IFC) speed discrimination task. They had to discriminate the motion speed between two motion displays undergoing similar motion by identifying which of the displays contained faster moving dots. Displays were randomly presented for 400 ms, 440 ms or 480 ms with a 300 ms inter-stimulus interval (Figure 2A). Optical flow stimuli of different types (expansion, contraction, rotation) were counterbalanced. Each type of motion display pair was presented 50 times. Subsequent to the presentation of each display pair, subjects were required to discriminate the speed of the two displays. If Display 1 was going faster they had to press one button and if Display 2 was faster they had to press the other button.

*Statistical methods description:* Speed discrimination thresholds were measured for a range of optic flow stimuli. Subjects were required to perform a 2IFC speed discrimination task between stimuli with similar motion types. Speed discrimination thresholds were assessed by running three adaptive staircase procedures (QUEST) in parallel, one for each motion type. The speed difference to which observers responded correctly with a probability of 75% was taken as the discrimination threshold. The mean thresholds of the three groups of subjects were compared by means of a one-way ANOVA for each optic flow type (Expansion, Contraction and Rotation).

#### Backward masking

*Stimuli*: Stimuli were displayed on the ViewSonic G90f+ with 1024×768 pixels, 100 Hz. A vertical vernier stimulus was presented consisting of two one-pixel wide segments each 616″ long and separated by a 137″-vertical gap. The two segments could be offset in the horizontal direction either to the left or to the right. Vernier offset was one pixel, i.e., 68″. It was immediately followed by a grating composed of five aligned verniers. Except for offset, spatial parameters of the vernier and the following grating elements were identical. The horizontal spacing between grating elements was 205″. The luminance of the stimuli was 100 cd/m^2^.

**Figure 3:**
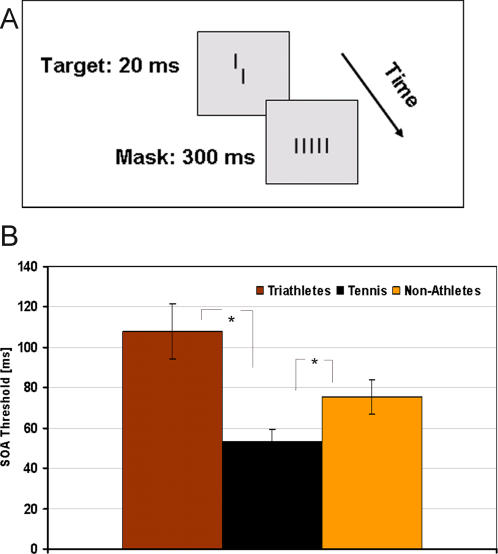
Backward masking. A: Stimuli of the backward masking task: The vernier was presented for 20 ms and the mask for 300 ms. The SOA was varied to find the threshold of each subject. B: Results of the backward masking task: The mean threshold is shown for each group of subjects. Error bars show standard errors.

*Procedure*: The vernier was presented for 20 ms and the grating lasted for 300 ms (Figure 3A). The ISI (inter-stimulus interval) varied but we plotted the SOA (Stimulus Onset Asynchrony), which is the ISI+vernier duration. The SOA was varied via an adaptive staircase procedure (PEST), with a starting value of 200 ms. PEST is an adaptive staircase procedure which tests the same value several times and then changes towards a shorter or longer SOA depending on the responses. In other words, the PEST procedure uses changes in step size to focus the adaptive track ever more finely. The PEST algorithm is designed to place trials at the most efficient locations along the stimulus axis in order to increase measurement precision while minimizing the number of trials required to estimate a threshold. We used the 75% correct value as threshold. There were 80 trials in total. Subjects had to indicate the horizontal displacement of the lower segment with respect to the upper one.

*Statistical methods description:* The 75% point of correct data was taken as a measure of discrimination threshold. The mean thresholds of the three groups of subjects were compared by means of a one-way ANOVA.

#### Tennis ball detection

**Figure 4:**
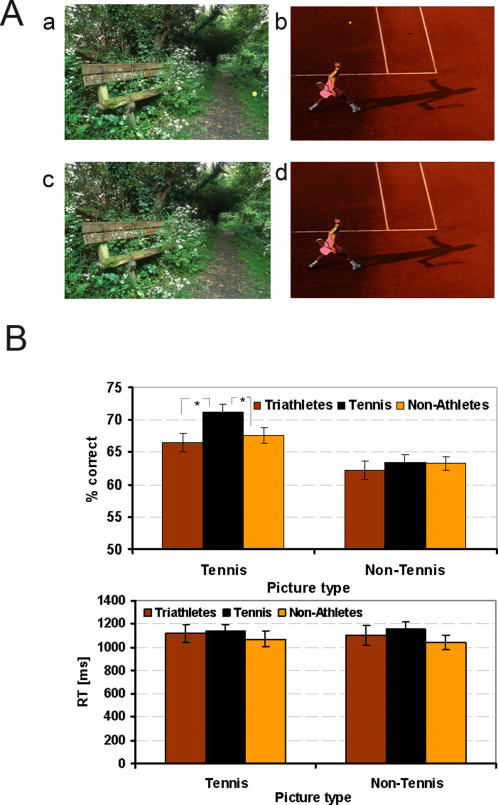
Ball detection. A: Examples of non-tennis pictures (a, c) and tennis related pictures (b, d) both with (a, b) and without (c, d) a tennis ball. B: Results of the ball detection task: Accuracy is shown in the top panel and reaction times are shown in the bottom panel for each picture type (tennis-related and non-tennis pictures). Error bars show standard errors.

*Stimuli*: Stimuli were displayed on the ViewSonic G90f+ with 1280×1024 Pixel, 75 Hz. This task was inspired by the study of Allard and Starkes [17] who showed volleyball players and non-players pictures of volleyball situations depicting either game or non-game situations. In the present study, colour pictures of tennis scenes (taken at the 2005 Roland Garros Grand Slam tournament) and non-tennis scenes were presented. The tennis pictures were typical game situations and the non-tennis pictures were scenes of landscapes and other sports games like soccer or volleyball (Figure 4A). The pictures' dimensions were 23×15 cm or 650×434 pixels.

*Procedure*: Pictures were presented individually on a computer screen for 13 ms. The same picture could appear several times, either with or without a tennis ball. There were 120 trials for each picture type (tennis and non-tennis related scenes), which were presented in separate blocks. Within each block, 60 pictures were presented with the ball and 60 without, in a random order. The blocks were counterbalanced between subjects. Subjects had to decide as quickly as possible whether a tennis ball was present or not by pressing either one of two push buttons.

*Statistical methods description:* Accuracy and speed were measured by calculating the correct percentage and mean RTs for each subject for tennis and non-tennis pictures over all trials. Accuracy and RTs performances of the three groups of subjects were compared by means of a one-way ANOVA for each picture type (tennis and non-tennis related pictures).

#### Pattern detection

**Figure 5:**
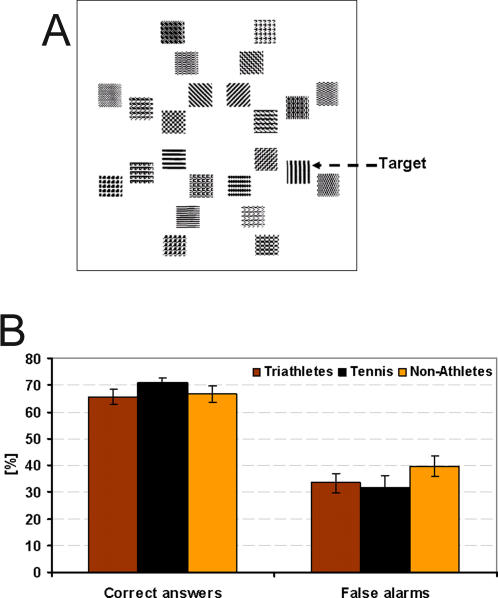
Pattern detection. A: Stimulus composed of 24 patterns. The target to be detected is shown by the arrow. B: Results of the pattern detection task: Accuracy is shown on the left part of the graph and false alarms are shown on the right part for each group of subjects. Error bars show standard errors.

*Stimuli*: Stimuli were displayed on the ViewSonic G90f+ with 1280×1024 Pixel, 75 Hz. In this task, we repeated the experiment of Buckles, Yund and Efron [25] who presented tennis players and non-players a group of patterns in which they had to detect a particular target. A typical example is shown in Figure 5A. The stimulus was composed of 24 patterns (size was approx. 60′×60′), 8 with their centres equally spaced on each of three concentric circles, the radii of which were 2.2°, 3.6° and 5.0°. The target to be detected was a vertical striped pattern.

*Procedure*: A fixation point was presented for a random time between 500 and 2000 ms before the stimulus, which was presented for 50 ms. There were 240 trials and the target could appear at any of the 24 locations (10 times at the same location). These were presented randomly. Within each trial, the locations of the non-target patterns were independently randomised. Subjects had to decide as quickly as possible whether the target was present or not by pressing either one of two push buttons. The target was present in 75% of the cases. In the remaining 25%, the target was replaced by another pattern.

*Statistical methods description:* Percent correct as well as false alarms were calculated. Accuracy performances and false alarms of the three groups of subjects were compared by means of a one-way ANOVA.

#### Attentional blink

**Figure 6:**
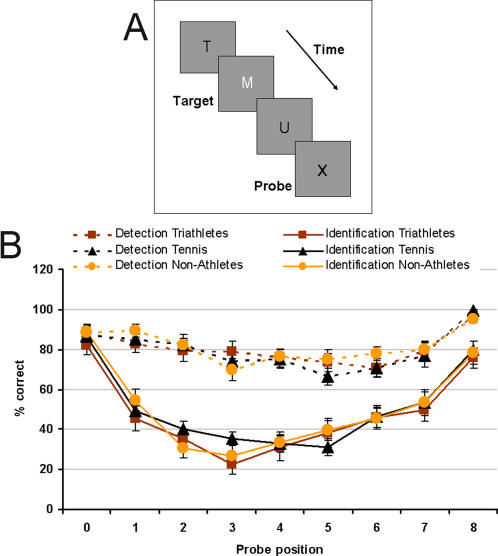
Attentional blink. A: Example of the attentional blink stimulus: A target (white letter) and a probe (letter “X”) were presented in a Rapid Serial Visual Presentation (RSVP) of black letters. B: Results of the attentional blink task: Accuracy of probe detection is shown on the top part of the graph for the control (detection) condition and on the bottom part of the graph for the experimental (identification) condition. Error bars show standard errors.

*Stimuli*: Stimuli were displayed on the ViewSonic G90f+ with 1280×1024 Pixel, 70 Hz. A target (white letter) and a probe (letter “X”) were presented within a Rapid Serial Visual Presentation (RSVP) of black letters (Figure 6A). That is, each trial consisted of a series of successively presented simple, block-style, upper case letters. Letters were 50′ in height the width was implicitly defined by the font. The experimental condition required subjects first to identify a white letter (target) embedded in a letter stream of black letters and subsequently to respond whether or not an “X” (probe) had been presented in the post-target letter stream. In the control condition, subjects were told to ignore the target and simply respond whether the “X” had been presented in the letter stream.

*Procedure*: 180 RSVP letter streams (trials) were presented. The computer randomly chose the letters to be presented from the 26 letters in the alphabet with the condition that no letter be presented twice within a trial. Each letter was presented for 14 ms with an inter-stimulus interval (ISI) of 71 ms. Each letter was displayed singly at the same location in the centre of a uniform grey field. The number of letters in the pre-target stream could vary between 7 and 15, however, the number of letters in the post-target stream was always 8. The probe “X” could appear at any of 9 positions, including the target position (in that case, the probe was presented in white) and the 8 following positions. The “X” was never presented prior to the target and never appeared twice within a single stream. The probe (“X”) was presented 10 times at each of the possible serial positions, yielding 90 probe-present trials. Subjects had to identify the target and detect the presence or absence of the probe. They identified the target by naming it (which was recorded by the experimenter) and responded for the presence or absence of the probe by pressing either one of two push buttons.

*Statistical methods description:* The percentage of trials in which the probe was correctly detected was calculated for both the experimental (identification) and control (detection) conditions. Percent correct performances of the three groups of subjects were compared by means of a two-way ANOVA for each task condition (identification and detection).

#### Flash lag

**Figure 7:**
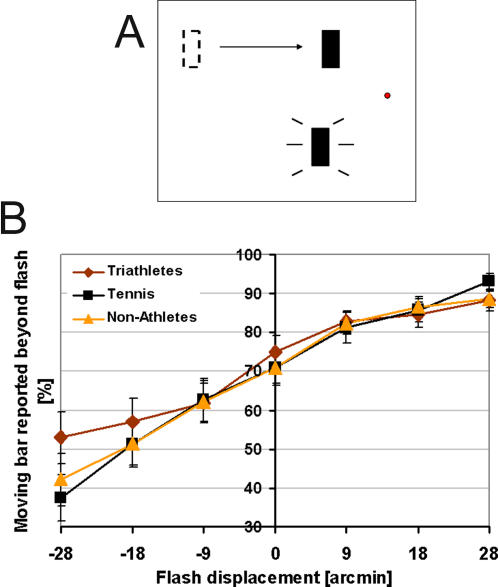
Flash-lag. A: Example of the flash-lag stimulus: A vertical bar was drifted horizontally towards a fixation point and a second vertical bar was flashed in synchrony with the last frame of the motion. B: Results of the flash-lag task: Proportion of trials of the moving bar reported as being beyond the flash as a function of flash displacement. Error bars show standard errors.

*Stimuli*: Stimuli were displayed on the ViewSonic G90f+ with 1280×1024 Pixel, 75 Hz. A vertical bar drifted horizontally towards a fixation point and a second vertical bar was flashed in synchrony with the last frame of the motion (Figure 7A). Moving and flashed bars were the same size (approx. 160′×16′).

*Procedure*: The moving bar was presented for 520 ms and its speed was 16°/sec. The flash was synchronous with the last frame of the moving bar on all trials. Flash duration was 1 frame (13 ms). The horizontal position between the two bars varied from trial to trial using the method of constant stimuli. The vertical distance between the nearest edges of the bars (bottom edge of the top bar and top edge of the bottom bar) was 8.5°. The last frame of the moving bar was presented at a constant 3.92° horizontal distance from the fixation point, whereas the horizontal position of the flash relative to the last frame of the motion was varied between 0′, ±9.54′, ±19.08′, and ±28.62′. On half of all trials, the moving bar appeared in the upper visual field and the flashed bar in the lower visual field. This arrangement was reversed on the other half and all trials were randomly intermixed. There were 30 trials per condition (i.e., per horizontal position of the flash) leading to a total of 210 (30×7) trials per subject. Subjects had to judge the location of the moving bar with respect to the flashed bar after both bars had disappeared. In other words, in a binary task, observers had to judge whether the top or bottom bar was further right at the moment of the flash.

*Statistical methods description:* Accuracy performances of the three groups of subjects were compared by means of a one-way ANOVA for each task condition.

## Results

### Coherent motion

Figure 1B shows the mean accuracy percentage and mean RTs of the three groups of subjects for each level of coherence (5%, 10% and 15%).

The accuracy analyses for the three coherence levels failed to be significant (5% coherence: F(2, 52) = 1.19, p = .31, 10% coherence: F(2, 52) = 1.68, p = .19, and 15% coherence: F(2, 52) = 1.94, p = .15). Collapsing all three levels of coherence yielded a significant result (F(2, 52) = 6.09, p<.01; Figure 1C).

In the case of RTs, neither the individual (5% coherence: F(2, 52) = 1.39, p = .25, 10% coherence: F(2, 52) = 1.14, p = .32, and 15% coherence: F(2, 52) = .52, p = .59) nor the collapsed results (F(2, 52) = .18, p = .83; Figure 1C) yielded significant results. However, there was a speed-accuracy trade-off for tennis players versus non-athletes.

### Speed discrimination

Figure 2B shows the mean speed discrimination thresholds for each optic flow type and all three groups of subjects.

There was a significant effect for Expansion (F(2, 48) = 4.24, p<.05), with tennis players showing a lower threshold than triathletes and non-athletes. There was also a significant effect for Contraction (F(2, 47) = 5.33, p<.01). However, only the triathletes seemed to show a higher threshold than both the tennis players and the non-athletes (see Fig. 2). Further T-tests for independent samples showed a higher threshold for triathletes than tennis players (t(30) = 2.94, p<.01), while the lower threshold trend of tennis players compared to non-athletes failed to be significant (t(33) = −0.85, p = .39). Finally, the ANOVA for Rotation (F(2, 48) = .51, p = .60) was not significant.

### Backward masking

Figure 3B shows the mean threshold for all three groups of subjects.

There was a significant effect for threshold (F(2, 49) = 7.58, p<.01), revealing a lower threshold for tennis players than for triathletes and non-athletes.

### Ball detection

Figure 4B shows the mean accuracy percentage (Fig. 4B top) and mean RTs (Fig. 4B bottom) of all three groups of subjects.

Concerning accuracy performances, there was a significant effect for tennis-related pictures (F(2, 50) = 3.28, p<.05), with tennis players showing a higher percentage of correct answers compared to triathletes and non-athletes. Importantly, there was no significant effect for non-tennis pictures (F(2, 50) = .33, p = .72). Thus, tennis players were better than the two other groups of subjects at detecting a tennis ball in tennis-related pictures but not in non-tennis pictures.

For RTs performances, there were no significant effects for tennis-related pictures (F(2, 50) = .27, p = .76), nor non-tennis pictures (F(2, 50) = .71, p = .49).

### Pattern detection

Figure 5B shows the mean accuracy percentage and mean false alarm rate of all three groups of subjects.

There were no significant effects for accuracy (F(2, 51) = .96, p = .38), nor false alarms (F(2, 51) = 1.14, p = .32).

### Attentional blink

Figure 6B shows the mean percent correct for the detection and identification conditions in all three groups of subjects.

There were no significant effects for the detection condition (F(18, 84) = .90, p = .57), nor for the identification condition (F(18, 84) = 1.00, p = .46).

### Flash lag

Figure 7B shows the results of the spatial judgement task in percent of the moving bar reported as being beyond the flash in function of flash displacement.

Accuracy performances showed no significant effects (F(14, 90) = 1.4, p = .16). For all three groups of subjects, the perceived terminal position of the moving bar was beyond the perceived position of the flash, in the direction of the bar's motion. That is to say, there was a large lag-effect.

## Discussion

It has been shown that optometric vision does not improve with training [5], whereas cognitive skills, such as anticipation of opponents' intentions or recognition of typical patterns of play, do [1], [6]–[9].

The cognitive tasks used in sport research are generally sport-specific, related to a particular sport. For example, tennis players' visual anticipation skills are tested with videotapes of expert tennis players performing either serves or ground strokes [24]. In contrast, in the present study, our philosophy was rather in line with Buckles et al. [25], Morrillo et al. [26], and Di Russo et al. [27], [35] who used tasks unrelated to sport. Our tasks were rather perceptual than cognitive. In this sense, our tasks lay between the optometric and the cognitive ones. That is, they possibly involved higher level mechanisms than the optometric tasks but lower level mechanisms than the cognitive tasks. Our goal was to determine whether at this level we would find some basic visual perceptual skills which improved and others which did not in tennis players.

We investigated differences between tennis players, triathletes, and non-athletes on seven visual tasks covering a wide range of perceptual functions including motion and temporal processing, object detection, and attention.

First, we tested the participants' sensitivity to coherent motion within randomly moving dots. We expected tennis players to perform better in this task than triathletes and non athletes since tennis players need to focus on ball trajectories. Indeed, tennis players were more accurate, however, without being faster than both control groups. Therefore, tennis players seem to have an accuracy advantage over other subjects in detecting movement. This result is in accordance with Williams et al. [36] who showed that skilled tennis players were able to follow the flight of a ball more smoothly than their less skilled counterparts. Whereas Williams et al. [36] used a specific tennis-related task, we used an unspecific one. Therefore, in contrast to Williams et al. [36], we detected a fundamental skill that seems to be influenced by tennis, free of tennis context. However the data needs to be taken with caution because of a speed-accuracy trade-off.

This finding was corroborated and extended in the speed discrimination task, where tennis players performed better than both controls and triathletes. Yet, this was only found for the expanding movement of dots (i.e., dots approaching participants) and not for the contracting or the rotating movements of dots. This could be related to the fact that tennis players are used to seeing tennis balls approaching them at high speeds. Thus, it shows that only basic skills that relate to tennis seem to improve in tennis players. This is in agreement with Moreno et al. [37], who also observed increased performance in experienced players when judging a frontal trajectory (approaching the observer) compared to other trajectories. However, whereas Moreno et al. [37] used a specific task with real tennis balls, we identified a fundamental skill that again seems to be influenced by tennis.

Backward masking was used to study general temporal processing differences between tennis players and controls in time limited, partly unconscious, situations (review about masking: [31]). We hypothesized that tennis players would perform better in this task than triathletes and non-athletes because of the superior temporal processing supposedly acquired during tennis training and also because information is often not processed consciously in sport games when visual information must be processed under strong time constraints [38]. We indeed observed better performance in tennis players than in both control groups, which might indicate that tennis players perform better in general than triathletes and non-athletes under conditions of strong time limitations.

So far, most of the temporal tasks we used all seemed to reveal some advantage for tennis players over triathletes and non-athletes. In the object-related tasks, the results were somewhat more balanced. There was a significant advantage for tennis players over triathletes and non-athletes in the detection of tennis balls, but only when presented in the context of tennis (ball detection task). Therefore, it seems that the familiar context facilitates visuo-spatial processing in athletes. This task was the only sport-specific task we used. The results observed are in line with many of the sport-specific cognitive tasks and namely with the study of Allard and Starkes [17] who required subjects to detect a volley ball in photographs presented tachistoscopically for only 17 ms. Highly experienced volleyball players detected the ball in these photographs faster and more precisely than non-players. In our case, tennis players were more accurate but not faster than the other participants in detecting the tennis balls. This effect of context familiarity has also been shown in recall tasks using sport-specific stimuli. For example, Allard, Graham and Paarsalu [39] showed that basketball players were superior to non-players in remembering the position of basketball players after a 4-second view of a slide of a structured game situation. However, players and non-players did not differ in the recall of unstructured game situations.

In the pattern detection task, the slight advantage of tennis players over the other participants was not significant. A possible explanation could be that, unlike the ball detection task, this pattern detection task was less tennis specific and therefore no familiar context facilitation effect, as discussed above, could occur. Surprisingly, we were not able to replicate the results of Buckles, Yund and Efron [25] who had found an advantage for tennis players over non-players using the same task (however, our study had less observers and hence, less statistical power).

Regarding the attentional tasks, the attentional blink was chosen because it involves rapid changes in visual information, and blanking out information that might be problematic for tennis players. Therefore, we supposed that tennis players would present an advantage in this task over the other groups of participants because they may be able to allocate their attention more effectively (due to rapid changes in tennis game situations). However, no significant differences were found in this task. While many studies have shown the importance of attention in sports [40]–[42], they all used a spatial cuing paradigm. By using another attentional task than spatial cuing, we attempted to provide a more complete picture of attentional orienting in athletes, as suggested by McAuliffe [43]. However, our task did not differentiate our populations and, therefore, it seems that attention as assessed by the attentional blink is not a fundamental skill that is particularly improved in tennis compared to other sports and in general.

As mentioned in the introduction, the flash-lag task can be related to attention (the observer's attentional set contributes to the modulation of perceptual latencies involved in the alignment task; [44]) and motion extrapolation. We expected tennis players to perform better in this task because of the need of anticipation of the ball trajectory. This was supported by Moreno et al. [37], who suggested that experienced athletes have a more precise perception of the trajectory of moving objects than non-athletes. However, the flash-lag task did not yield significant results. The kind of attention measured with this task might not be a fundamental skill particularly improved in tennis players compared to other athletes.

To summarize, we indeed found that some perceptual skills improve with tennis whereas others do not. Our results suggest that speed processing and temporal processing is often faster and more accurate in tennis players, particularly, when dots are expanding (speed discrimination) or under strong time constraints (backward masking). Hence, it seems that either playing tennis improves temporal processing or better tennis playing is caused by better temporal processing, or any combination of both. Object detection tasks only showed an advantage for tennis players in context dependent situations. Although attention is certainly an important skill in tennis, the types of attention we tested did not reveal any differences among our populations, and, therefore, did not transfer to fundamental skills. Thus, based on the current selection of tasks, temporal processing seems to be what is mostly required for and reinforced in tennis skills, at least with respect to the test paradigms that we have used in the present study. However, the effects found were rather small and this can be explained by several reasons. First, normal subjects usually show performance close to ceiling because of everyday demands such as car driving. Therefore, if the fundamental skills change in athletes, we can only expect small effects. Secondly, it is a priori unclear *which* fundamental perceptual tasks will show differences between athletes and non-athletes.

Therefore, it is rather (positively) surprising that even with a small sample of subjects we found significant differences. One could argue that all the effects found are related to any sport training or some general level of eagerness and not to tennis. This is precisely why we added the group of triathletes as controls because they train as hard as tennis players but have lower visual processing demands in their sport. Our data show that general physical training or eagerness are not sufficient to account for the effects observed in tennis players suggesting that the present effects can be linked to tennis (and possibly to other racket/ball sports).

Finally, a cautionary remark seems mandatory. By breaking down complex tasks into simple visual perception tasks, the present study has allowed identifying some important visual mechanisms in tennis players. Yet, we see the study as a starting point for future, larger studies in the field applying fundamental tasks to larger and independent samples. As mentioned in the introduction, visual functions were previously generally examined either via optometry (which are known to be tasks that cannot be learned) or via sport-specific cognitive tasks. Our approach was to identify which fundamentally *less unspecific* perceptual tasks are improved by decades of tennis training. We believe that many factors contribute to the development of visual-perceptual skill and that fundamental (unrelated to sport) visual skills are one of them.

Certain motion detection, speed discrimination, and backward masking are basic perceptual skills in which tennis players were superior. The question is whether training with these tasks can improve tennis performance. Training of specific cognitive tasks has been shown to improve visual search in tennis [45], [46]. Similarly, it has been shown that specific and extensive practice can lead to neural economy of motor preparation and modification of elementary visuo-motor functions [35], [47]. Therefore, just like visual search and visuo-motor functions can be improved with training, we suggest that the basic visual skills of motion detection or speed discrimination can be improved with training as well and might lead to improved tennis performance.

## References

[1] Helsen WF, Starkes JL (1999). A multidimensional approach to skilled perception and performance in sport.. Appl Cogn Psychol.

[2] Coffey B, Reichow AW, Loran DFC, MacEwen CJ (1995). Visual performance enhancement in sport optometry.. Sports vision.

[3] Loran DFC, Griffiths GW (1998). Visual performance and soccer skills in U14 players.. SVA Newsletter Issue.

[4] Westheimer G (2001). Is peripheral visual acuity susceptible to perceptual learning in the adult?. Vision Res.

[5] Abernethy B, Wood JM (2001). Do generalized visual training programmes for sport really work? An experimental investigation.. J Sports Sci.

[6] Starkes J (1987). Skill in field hockey: the nature of the cognitive advantage.. J Sport Psychol.

[7] Abernethy B, Neal RJ, Koning P (1994). Visual-perceptual and cognitive differences between expert, intermediate and novice snooker players.. Appl Cogn Psychol.

[8] Ward P, Williams AM (2003). Perceptual and cognitive skill development in soccer: the multidimensional nature of expert performance.. J Sport Exerc Psychol.

[9] Starkes JL, Helsen W, Jack R, Singer RN, Hausenblas HA, Janelle CM (2001). Expert performance in sport and dance.. Handbook of sport psychology.

[10] Abernethy B, Russell DG (1987). The relationship between expertise and visual search strategy in a racquet sport.. Hum Mov Sci.

[11] Williams AM, Burwitz L, Reilly T, Clarys J, Stibbe A (1993). Advance cue utilization in soccer.. Science and football II.

[12] Williams AM, Davids K (1998). Visual search strategy, selective attention, and expertise in soccer.. Res Q Exerc Sport.

[13] Williams AM, Davids K, Burwitz L, Williams JG (1994). Visual search strategies in experienced and inexperienced soccer players.. Res Q Exerc Sport.

[14] Williams AM, Davids K (1995). Declarative knowledge in sport: A byproduct of experience or a characteristic of expertise.. J Sport Exerc Psychol.

[15] Williams AM, Davids K, Burwitz L, Williams JG (1993). Cognitive knowledge and soccer performance.. Percept Mot Skills.

[16] Abernethy B, Russell D (1984). Advance cue utilization by skilled cricket batsmen.. Australian J Sci Med Sport.

[17] Allard F, Starkes L L (1980). Perception in sport: volleyball.. J Sport Psychol.

[18] Borgeaud P, Abernethy B (1987). Skilled performance in volleyball defense.. J Sport Psychol.

[19] Didierjean A, Marmèche E (2005). Anticipatory representation of visual basketball scenes by novice and expert players.. Vis Cogn.

[20] French KE, Thomas JR (1987). The relation of knowledge development in children's basketball performance.. J Sport Psychol.

[21] Goulet C, Bard C, Fleury M (1989). Expertise differences in preparing to return a tennis serve: a visual information processing approach.. J Sport Exerc Psychol.

[22] McPherson SL, Thomas JR (1989). Relation of knowledge and performance in boys' tennis: age and expertise.. J Exp Child Psychol.

[23] Starkes J, Allard F, Lindley S, O'Reilly K (1994). Abilities and skill in basketball.. Int J Sport Psychol.

[24] Singer RN, Cauraugh JH, Chen D, Steinberg GM, Frehlich SG (1996). Visual search, anticipation, and reactive comparisons between highly-skilled and beginning tennis players.. J Appl Sport Psychol.

[25] Buckles KM, Yund EW, Efron R (1991). Visual detectability gradients: effect of high-speed visual experience.. Brain Cogn.

[26] Morrillo M, Di Russo F, Pitzalis S, Spinelli D (2006). Latency of prosaccades and antisaccades in professional shooters.. Med Sci Sports Exerc.

[27] Di Russo F, Pitzalis S, Spinelli D (2003). Fixation stability and saccadic latency in elite shooters.. Vis Res.

[28] Lees A (2003). Science and the major racket sports: a review.. J Sports Sci.

[29] Schulte-Körne G, Bartling J, Deimel W, Remschmidt H (2004). Visual evoked potentials elicited by coherently moving dots in dyslexic children.. Neurosci Letters.

[30] Clifford CWG, Beardsley SA, Vaina LM (1999). The perception and discrimination of speed in complex motion.. Vision Res.

[31] Breitmeyer BG, Ogmen H (2006). Visual Masking: Time slices through conscious and unconscious vision..

[32] Raymond JE, Shapiro KL, Arnell KM (1992). Temporary suppression of visual processing in an RSVP task: an attentional blink?. J Exp Psychol: HPP.

[33] Kanai R, Sheth BR, Shinsuke S (2004). Stopping the motion and sleuthing the flash-lag effect: spatial uncertainty is the key to perceptual mislocalization.. Vision Res.

[34] Bach M (1996). The Freiburg Visual Acuity test - automatic measurement of visual acuity.. Optom Vision Sci.

[35] Di Russo F, Taddei F, Aprile T, Spinelli D (2006). Neural correlates of fast stimulus discrimination and response selection in top-level fencers.. Neurosci Letters.

[36] Williams AM, Singer RN, Weigelt C, Lees A, Maynard I, Hughes M, Reilly T (1998). Visual search strategy in „live“ on-court situations in tennis.. Science and Racket Sports II.

[37] Moreno FJ, Luis V, Salgado F, Garcia JA, Reina R (2005). Visual behaviour and perception of trajectories of moving objects with visual occlusion.. Percept Mot Skills.

[38] Kibele A (2002). Bewegungspriming. Implizit erlernte Reaktionshandlungen im Spiel- und Zweikampfsport, wobei die auszulsenden visuellen Reize nicht diskriminiert zu werden brauchen.. Leistungssport.

[39] Allard F, Graham S, Paarsalu M (1980). Perception in sport: basketball.. J Sport Psychol.

[40] Casteillo U, Umiltà C (1992). Orienting of attention in volleyball players.. International J Sport Psychol.

[41] Enns J, Richards J (1997). Visual attentional orienting in developing hockey players.. J Exp Child Psychol.

[42] Nougier V, Ripoll H, Stein JF (1989). Orienting of attention with highly skilled athletes.. Int J Sport Psychol.

[43] McAuliffe J (2004). Differences in attentional set between athletes and nonathletes.. J Gen Psychol.

[44] Namba J, Baldo MVC (2004). The modulation of the flash-lag effect by voluntary attention.. Perception.

[45] Farrow D, Abernethy B (2002). Can anticipatory skills be learned through implicit video-based perceptual training?. J Sports Sci.

[46] Williams AM, Ward P, Knowles JM, Smeeton NJ (2002). Anticipation skill in a real-world task: measurements, training, and transfer in tennis.. J Exp Psychol: Appl.

[47] Di Russo F, Pitzalis S, Aprile T, Spinelli D (2005). Effect of practice on brain activity: an investigation in top-level rifle shooters.. Med Sci Sports Exerc.

